# Assessment of genetic changes and neurovirulence of shed Sabin and novel type 2 oral polio vaccine viruses

**DOI:** 10.1038/s41541-021-00355-y

**Published:** 2021-07-29

**Authors:** Rahnuma Wahid, Laina Mercer, Andrew Macadam, Sarah Carlyle, Laura Stephens, Javier Martin, Konstantin Chumakov, Majid Laassri, Svetlana Petrovskaya, Saskia L. Smits, Koert J. Stittelaar, Chris Gast, William C. Weldon, Jennifer L. Konopka-Anstadt, M. Steven Oberste, Pierre Van Damme, Ilse De Coster, Ricardo Rüttimann, Ananda Bandyopadhyay, John Konz

**Affiliations:** 1 Center for Vaccine Innovation and Access, PATH, Seattle, WA USA; 2 National Institute for Biological Standards and Control (NIBSC), Hertfordshire, UK; 3 Center for Biologics Evaluation and Research, Food and Drug Administration, Silver Spring, MD USA; 4 Global Virus Network Center of Excellence, Baltimore, MD USA; 5 Viroclinics Biosciences B.V., Rotterdam, the Netherlands; 6 Viroclinics Xplore, Viroclinics Biosciences B.V., Rotterdam, the Netherlands; 7 Division of Viral Diseases, Centers for Disease Control and Prevention, Atlanta, GA USA; 8 Centre for the Evaluation of Vaccination, Vaccine and Infectious Disease Institute, University of Antwerp, Antwerp, Belgium; 9 Fighting Infectious Diseases in Emerging Countries (FIDEC), Miami, FL USA; 10 Bill and Melinda Gates Foundation, Seattle, WA USA

**Keywords:** Policy and public health in microbiology, Public health, Live attenuated vaccines, Viral evolution

## Abstract

Sabin-strain oral polio vaccines (OPV) can, in rare instances, cause disease in recipients and susceptible contacts or evolve to become circulating vaccine-derived strains with the potential to cause outbreaks. Two novel type 2 OPV (nOPV2) candidates were designed to stabilize the genome against the rapid reversion that is observed following vaccination with Sabin OPV type 2 (mOPV2). Next-generation sequencing and a modified transgenic mouse neurovirulence test were applied to shed nOPV2 viruses from phase 1 and 2 studies and shed mOPV2 from a phase 4 study. The shed mOPV2 rapidly reverted in the primary attenuation site (domain V) and increased in virulence. In contrast, the shed nOPV2 viruses showed no evidence of reversion in domain V and limited or no increase in neurovirulence in mice. Based on these results and prior published data on safety, immunogenicity, and shedding, the nOPV2 viruses are promising alternatives to mOPV2 for outbreak responses.

## Introduction

Despite tremendous progress in the reduction of the incidence of poliomyelitis, global eradication remains elusive. One of the challenges is the genetic instability of the Sabin oral polio vaccine (OPV) strains, which can lead to vaccine-associated paralytic polio (VAPP) in recipients or their immediate contacts and, in regions with persistently under-immunized populations, virulent circulating vaccine-derived poliovirus (cVDPV) strains that cause outbreaks. Certification of eradication of wild type 2 poliovirus in 2015 and the known risks of VAPP and cVDPV led to cessation of routine use of the type 2 component of Sabin OPV in 2016, with a coordinated switch from trivalent to bivalent OPV in OPV-using countries. Since the switch, cVDPV2 outbreaks have occurred with increasing frequency and a number of cVDPV2-associated paralysis cases have been identified in 2019 and 2020 in a broader range of geographies^1^. Furthermore, evidence indicates that in some cases, the use of mOPV2 to combat outbreaks is seeding outbreaks in adjacent areas^2,3^.

The genetic evolution of the three Sabin serotypes (1, 2 and 3) which underlies the loss of attenuation during replication has been studied extensively. For all three strains, polymorphisms in domain V of the 5′ untranslated region are critical to reacquisition of virulence^4–6^. For Sabin 2, the early steps of the loss of attenuation involve nucleotide changes A481G in domain V, U398C in domain IV, and polymorphisms in the codon for amino acid 143 of the VP1 capsid protein (VP1-143) that usually exchange isoleucine for valine or threonine^6^. The A481G reversion happens quickly, with most samples being at least partially reverted by 5-7 days post-vaccination^7,8^.

A number of groups independently worked to design new replicating, attenuated virus strains that could potentially replace the Sabin strains for vaccination^9–14^. In 2011, a research consortium initiated efforts to combine different approaches to stabilize the attenuation, ultimately leading to selection of two novel OPV2 (nOPV2) candidates for clinical development^15,16^. In both candidates, domain V is genetically stabilized through the replacement of all U–G pairs with C–G or U–A pairs while maintaining a similar thermostability of the RNA structure as the Sabin strain (Supplementary Fig. 1a)^11^.

For one strain, nOPV2 candidate 1 (nOPV2-c1), domain V stabilization is coupled with relocation of the cis-acting recombination element (cre) and mutations to the 3D polymerase. Relocation of the cre element (cre5, Supplementary Fig. 1b) into the 5′ untranslated region ensures that the stabilized domain V cannot be replaced by a single 5′ recombination event, since the product of such an event would lack the essential cre element. The mutations to the 3D polymerase were selected to enhance fidelity (Hifi3, D53N) and reduce recombination (Rec1, K38R) events [10, 11]. The nOPV2 candidate 2 (nOPV2-c2) combines domain V stabilization with synonymous codon deoptimization in the capsid region [12]. Codon deoptimization leading to an increased number of CpG dinucleotides has been shown to provide further attenuation and reduce replicative fitness, which could also reduce transmission risk^9,10^. Promising in-human safety, immunogenicity, shedding, and high-level genetic stability results for these candidates were recently published^17–19^.

Here we describe development of the genetic and phenotypic methods to evaluate and compare novel OPV2 and Sabin 2 viruses shed by vaccinees. The results demonstrate the suitability of the methods to readily detect reversion mutations and discriminate shed nOPV2 from shed mOPV2 strains, affirming the potential of these novel strains.

## Results

### Method development and qualification

**Fig. 1:**
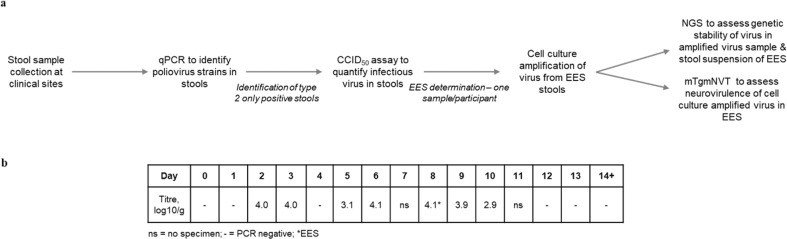
Overview of stool sample analysis and EES identification. **a** Genetic stability workflow. **b** EES selection. Hypothetical CCID_50_/g data from a clinical participant, to demonstrate selection of the EES.

Given the unique nature of the novel OPV2 strains, we reasoned that a convincing demonstration of differentiation of shed nOPV2 from shed mOPV2 strains would require a phenotypic measure of neurovirulence as well as supportive assessment of genetic heterogeneity leading to a workflow using mouse neurovirulence and next-generation sequencing (NGS) (Fig. 1). In this workflow, a single specimen (the Exploratory Endpoint Specimen, EES) is selected from the viral shedding time course. The EES is the latest sample that can reliably be analyzed by both methods. As noted in Fig. 1, the mouse neurovirulence test requires culture amplification from the stool to achieve the required potencies. Therefore, method development focused on (1) understanding the performance of the NGS method as applied to the stool extracts and culture-amplified virus, (2) selecting the conditions and dose for the mouse neurovirulence test, and (3) establishing the minimum virus concentration in stool that could be amplified in cell culture with minimal bias.

### Next-generation sequencing method characterization

Previous work^20,21^ identified the benefit of incorporating full-length genome amplification in advance of next-generation sequencing (NGS) to analyze shed poliovirus in stool extracts. To qualify the poliovirus full-length genome amplification and NGS pipeline, a research stock of Sabin 2 and stools from a prior clinical trial^22^ with Sabin 2 at varying titres were used.

Specificity and repeatability of the genome amplification and subsequent NGS were determined in triplicate analyses of a dilution series of Sabin 2 virus stock and Sabin 2-containing stool samples. The reverse transcription and DNA amplification procedure consistently produced an amplicon of appropriate size (~7.5 kb at ≥0.2 ng/µl) when titres were ≥4 log_10_ CCID_50_/gram of stool while all negative controls did not produce amplicons. Greater than 95% of NGS reads mapped to the Sabin 2 reference sequence in poliovirus-positive samples (data not shown). Similar results were obtained for nOPV2-c1 and nOPV2-c2 virus stocks when mapped to the relevant nOPV2-c1 or nOPV2-c2 reference.

### Evaluation of amplification conditions to determine minimum analyzable virus titre in stool

**Fig. 2:**
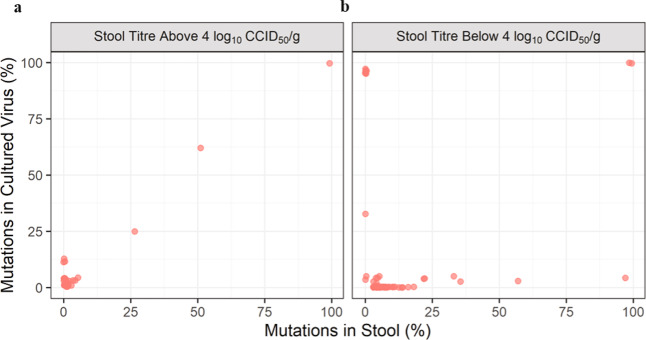
Comparison of frequency of polymorphisms present in stools and culture-amplified virus isolates from four participants administered mOPV2. Stools were collected at different times post vaccine administration. **a** Stool samples with viral titre above 4 log CCID_50_/g versus virus amplified in cell culture from those stool samples. **b** Stool samples with titre below 4 log CCID_50_/g versus virus amplified in cell culture from those samples.

In order to evaluate a 10^4^ CCID_50_ test sample dose in 5 µl in the mTgmNVT (the rationale for which is explained below), a titre of 6.3 log_10_ CCID_50_/ml is required for the mouse injections. Such titres were not anticipated in stool samples collected weeks after vaccination, thereby necessitating culture amplification. Low-temperature amplification conditions were used to avoid confounding the known temperature-sensitive phenotype of Sabin 2 and the candidate strains. HEp-2C cells were identified as a permissive cell line known to place relatively low selective pressure on poliovirus, and were infected at 33 °C for viral amplification^23^. The selected culture conditions, including the minimum titre which could be reliably amplified, were confirmed by comparing NGS results on shed virus samples from four mOPV2 recipients^22^ with amplified virus from the same stools. Figure 2a shows that for stool samples with virus titre above 4 log_10_ CCID_50_/gram, there was strong correlation between the variants observed directly in stool versus amplified virus. Correlation data as a function of stool titre are provided in Supplementary Table 2. For samples with titres lower than below 4 log_10_ CCID_50_/gram (Fig. 2b) the correlation was poor, with some polymorphisms detected in the stool samples not observed in cultured virus, and vice versa. The threshold for EES selection was therefore set at 4 log CCID_50_/gram based on these results.

### Development of a single-dose mouse neurovirulence test

The oral polio vaccine mouse neurovirulence release test (TgmNVT)^24^, which requires 64 mice each per sample and control (Supplementary Table 1) was modified into a higher-throughput format that could readily detect reversion of type 2 viruses. To select both the dose level of inoculum and the number of mice to be tested per sample, data were collected from experiments involving shed Sabin 2 virus samples from a previous clinical trial^22^ as well as cell culture passaged nOPV2-c2 virus evaluated in two poliovirus receptor (PVR) transgenic mouse lines: TgPVR21 (used in the vaccine release test) and Tg66 (laboratory research mouse line developed at NIBSC). The nOPV2-c2 virus was chosen because it is the less attenuated of the two vaccine candidates in mouse models, and would therefore be more difficult to distinguish from Sabin 2 than nOPV2-c1.

**Fig. 3:**
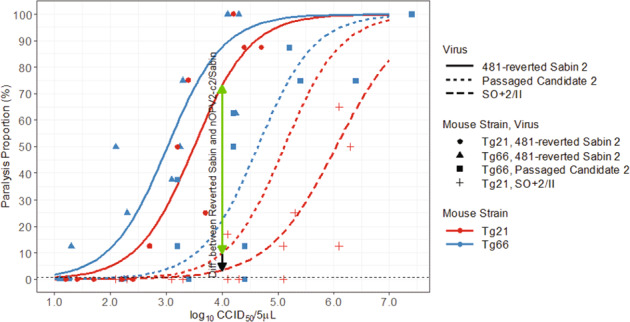
Selection of dose level of inoculum used in the modified mouse neurovirulence test. Estimated/predicted probability of mouse paralysis (y-axis) as a function of inoculum (*x*-axis), virus (dashed versus solid lines), and mouse strain (Tg21 versus Tg66), shown overlaid on data used to fit the logistic regression model. Model fit to data with Tg66 mice (passaged nOPV2-c2 and 481-reverted Sabin 2) and with Tg21 mice (481-reverted Sabin 2 only), and used to predict curve for Tg21 mice with passaged nOPV2-c2 inoculum. Vertical lines and associated annotations denote the difference in estimated paralysis rate between passaged nOPV2-c2 and 481-reverted Sabin 2 (solid green), and also between non-reverted Sabin 2 (SO + 2/II) and a 481-reverted Sabin 2 (solid green + solid black extension) in Tg21 strain mice at the nominal inoculum selected (4.0 log_10_ CCID_50_/5 µl) to enable differentiation of paralysis rates.

A binomial logistic generalized linear regression model was fitted to data from each intraspinal injection experiment, yielding an estimate of mouse paralysis proportion as a function of inoculum. These results are summarized in Fig. 3. Simulations based on this model fit were conducted to evaluate and select a dose level and number of mice per EES required to have sufficient statistical power to discriminate the paralysis rate of shed candidate virus samples from shed Sabin 2 samples at a single fixed dose of inoculum, which resulted in selection of a 10^4^ CCID_50_ dosage and ten mice per sample. The number of mice per control and assay validity criteria were established by resampling and simulating from de-identified historical data from prior lot-release assay runs of the WHO TgmNVT. A detailed comparison of this modified method (mTgmNVT) and the WHO TgmNVT for lot release is provided in Supplementary Table 1.

The finalized workflow for sample identification, NGS, culture-amplification, and mouse neurovirulence testing was then applied for analysis of nOPV2 and mOPV2 control clinical trials.

### Next-generation sequencing evaluations of shed virus

The workflow described in Fig. 1a was applied to samples collected from 1- to 5 year-old children in Lithuania who had previously received 3 or more doses of IPV, had no documented prior OPV exposure, and were subsequently vaccinated with mOPV2 during a phase 4 trial (M3 study, described in METHODS).

**Fig. 4:**
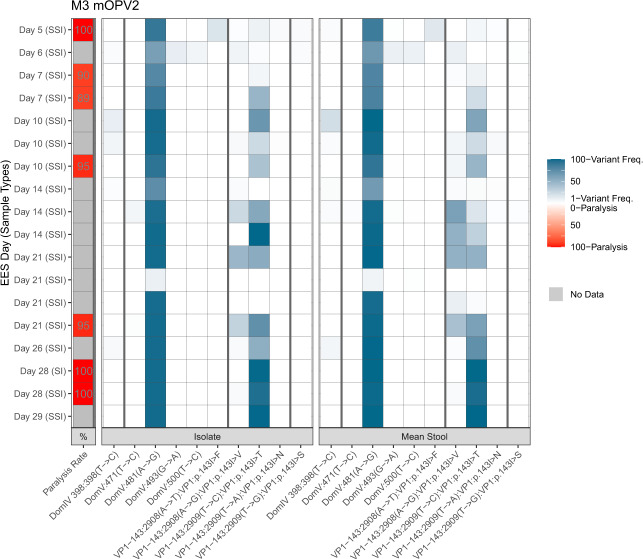
mOPV2. Frequency of variants in EES at known attenuation sites and mTgmNVT results. Data from M3 phase 4 trial. EES day shown with mTgmNVT result (red colour gradient) and variant frequency (blue colour gradient) averaged across two stool replicates (SS) and culture-amplified virus (I), if present. Amino acid associated with variant indicated, if applicable. S = Stool replicate; I = Culture-amplified virus isolate (SSI = Two stool replicates and culture-amplified virus isolate; SI = Single stool replicate and isolate). White cells = variant not detected. Grey cells = mTgmNVT result not available. The NGS pipeline reports the variants as SNPs. Coding impact assumes changes are not in common genomes when multiple variants are observed in VP1-143. All variants reported have Quality scores (Q scores) ≥ 30.

A subset of twenty participants who had provided stool samples on at least days 7, 14, 21, and 28 post-vaccination and had a sample meeting the EES criteria were selected for evaluation. The twenty EES ranged from 5 to 29 days post-vaccination. Eighteen of these EES were successfully sequenced, with two excluded due to insufficient DNA concentration following NGS library preparation. As expected for an RNA virus, there were several variants observed throughout the genome at varying frequencies (Supplementary Tables 3, 4). The assessment was focused on variants known to be associated with viral adaptation and virulence. In Figs. 4, 7a, b, the average frequency of polymorphisms of the two NGS replicates from the stool extract are shown for three important loci associated with loss of attenuation: position 398 in domain IV, position 481 in domain V, and in positions 2908 and 2909 (i.e. polymorphism frequencies residing in the codon for amino acid VP1-143).

The U398C polymorphism was observed in 7 of the 18 samples at values ranging from 1% to 25%, with limited relationship between the collection day and extent of change observed.

The A481G reversion at the primary attenuation site occurred rapidly, with 17 of 18 samples showing consensus-level reversion. The mean frequency of 481G was 87%, with a range of 7–99.7%.

Variants in amino acid VP1-143, the secondary attenuation site, were also commonly observed, with a trend of increasing total polymorphism frequencies in later EES. Five distinct polymorphisms were observed: A2908G, A2908T, T2909A, T2909C, and T2909G, with 2908G and 2909C being most common and resulting in change of isoleucine to valine or threonine, respectively. For EES with multiple polymorphisms in the VP1-143 codon, co-location of SNPs (single nucleotide polymorphisms) was assessed in a supplemental analysis of the FASTQ files in order to understand the coding impact. No co-location of polymorphisms above 1% frequency were observed in this re-analysis.

### Phase 1 and 2 trials with nOPV2-c1

nOPV2-c1 was administered to 15 and 17 adult participants with an exclusively IPV vaccination history in phase 1 (M4a) and phase 2 (M4) trials, respectively. For all 15 participants in M4a an EES was available, with collection dates ranging from 2 to 56 days post-vaccination. Eight EES were identified from the M4 trial with collection dates from 3 to 21 days post-vaccination. NGS was performed on both stool suspension (in duplicate) and culture-amplified virus and variants in modified regions are reported in Supplementary Tables 5 and 7. Variants outside of the modified regions are reported in Supplementary Tables 6, 8. Some of these variants [e.g. VP1 G3425A (E295K), VP1 A3053G (N171D) and VP4 C930T (A41V)] are present in the vaccine lots. Others appear in one or two EES and are not known to be associated with loss of attenuation.

**Fig. 5:**
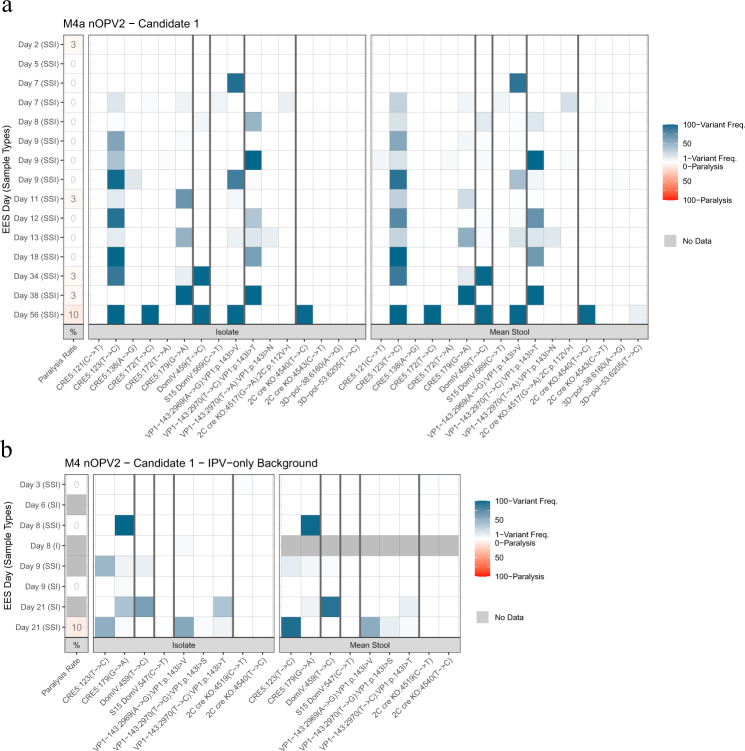
nOPV2-c1. Frequency of variants in EES at known attenuation sites and mTgmNVT results. Participant vaccination history is IPV only. EES day shown with mTgmNVT result (red colour gradient) and variant frequency (blue colour gradient) averaged across two stool replicates (SS) and culture-amplified virus (I), if present. Amino acid associated with variant indicated, if applicable. S = Stool replicate; I = Culture-amplified virus isolate (SSI = Two stool replicates and culture-amplified virus isolate; SI = Single stool replicate and isolate). White cells = variant not detected. Grey cells = mTgmNVT result not available or no NGS data from stool (S)/culture-amplified virus (I). The NGS pipeline reports the variants as SNPs. Coding impact assumes changes are not in common genomes when multiple variants are observed in VP1-143. **a** M4a phase 1 trial nOPV2-c1 cohort. **b** M4 phase 2 trial nOPV2-c1 cohort. All variants reported have Q scores ≥ 30.

Variants identified in the modified regions of the candidate virus or known to be associated with loss of attenuation for Sabin 2 are summarized in Figs. 5, 7 for these cohorts. Two polymorphisms in domain V were observed at low levels: C569U at approximately 6% in one EES from M4a, C547U at 1% in one replicate of an EES from M4.

Variants at two other sites implicated in viral adaptation and increases in virulence—U459C (U398C in Sabin 2) in domain IV and in VP1-143—were observed at levels comparable to those in Sabin 2^6^ (Figs. 5, 7b). The U459C polymorphism was observed at up to 93% in day 21 EES from M4 and up to 99% in day 34 and day 56 EES from M4a. VP1-143 variants were commonly observed in EES collected on or after day 7 in both trials, with a trend of increasing total polymorphism frequencies in later EES.

Polymorphisms at the base of the stem in the relocated cre5 at nucleotide 123 or 179 were observed in many of the samples and increased to 100% in later EES (Figs. 5, 7c). Positions 123 and 179 are opposite each other in a hairpin structure in a U–G pairing (Supplementary Fig. 1b). The mutations result in converting the U–G to either a stronger C–G or U–A pair. A single day 56 post-vaccination EES from M4a also showed 100% accumulation of a U172 C; this change would appear to increase the size of an internal loop in the cre5. In addition, two M4a samples accumulated a C121U polymorphism at <10%, which appears to extend the stem length by forming a U–A pair.

Low-level silent polymorphisms in the Rec1 and Hifi3 modifications were observed in day 9 and day 56 EES, respectively, from M4a. A number of variants were also observed in the cre knockout region (2C protease) in both trials and one polymorphism in particular (U4540C) accumulated to 100% in the day 56 EES from M4a.

Nine EES were identified from the 100 nOPV2-c1 recipients in M4 who had a history of OPV-vaccination. These EES were all collected on or before day 10 post-vaccination. NGS results for these samples are summarized in Fig. 7 and Supplementary Fig. 2a, with detailed results in Supplementary Tables 7, 8. Results for the modified regions and known attenuation sites were consistent with EES from similar collection days from participants with an IPV-only vaccination history.

### Phase 1 and 2 trials with nOPV2-c2

**Fig. 6:**
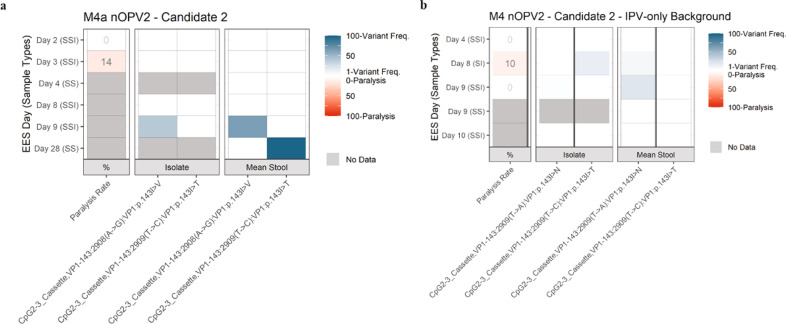
nOPV2-c2. Frequency of variants in EES at known attenuation sites and mTgmNVT results. Participant vaccination history is IPV only. EES day shown with mTgmNVT result (red colour gradient) and variant frequency (blue colour gradient) averaged across two stool replicates (SS) and culture-amplified virus (I), if present. Amino acid associated with variant indicated, if applicable. S = Stool replicate; I = Culture-amplified virus isolate (SSI = Two stool replicates and culture-amplified virus isolate; SI = Single stool replicate and isolate). White cells = variant not detected. Grey cells = mTgmNVT result not available or no NGS data from stool (S)/culture-amplified virus (I). The NGS pipeline reports the variants as SNPs. Coding impact assumes changes are not in common genomes when multiple variants are observed in VP1-143. **a** M4a phase 1 trial nOPV2-c2 cohort. **b** M4 phase 2 trial nOPV2-c2 cohort. All variants reported have Q scores ≥ 30.

In phase 1 (M4a) trial, 15 participants received nOPV2-c2. EES were identified for six participants with collection dates ranging from day 2 to day 28 post-vaccination. For the other nine participants, the virus titre in the stool never exceeded the minimum threshold for EES selection. Cell-culture amplification from the six EES yielded sufficient virus for NGS for only four participants. In phase 2 (M4) trial, five EES were identified from the 16 participants receiving nOPV2-c2, with collection day ranging from day 4 to 10 post-vaccination. Cell-culture amplification yielded sufficient virus for NGS for four of the five EES. The key variants observed are summarized in Fig. 6 and Supplementary Tables 9, 11. There were no reversions observed in the CpG-modifications of the P1 capsid region (Supplementary Tables 10, 12).

**Fig. 7:**
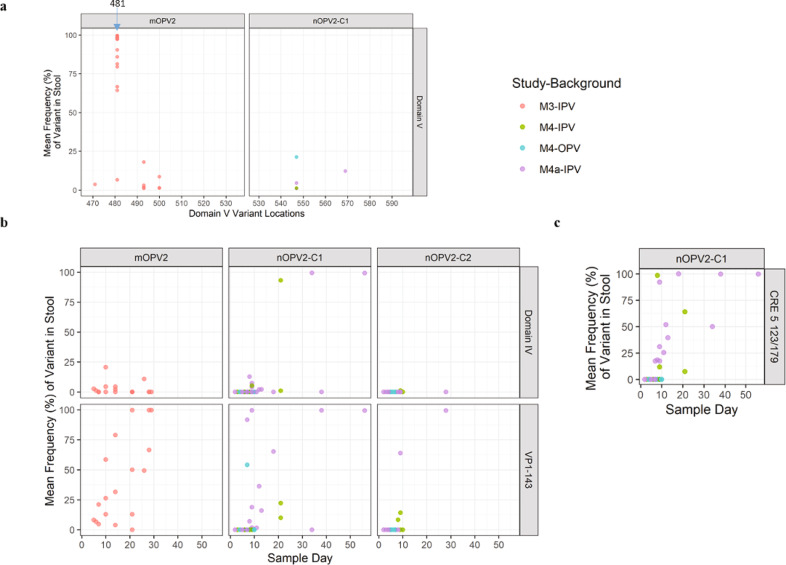
Frequency of key variants in EES by position or as a function of day post-vaccine administration. Mean frequencies of variants from two stools (if present) for each EES from each trial M4a, M4 and M3 are plotted. If variant is only present in 1 stool that value is presented. If variant is not present in either, assigned 0 for plotting purposes in **b** and **c**. **a** Domain V polymorphisms (468-535 for mOPV2/529-596 for nOPV2-c1) at each nucleotide position. **b** Domain IV (398 for mOPV2 and nOPV2-c2/459 for nOPV2-c1) and VP1-143 polymorphisms (2908-2910 for mOPV2 and nOPV2-c2/2969-2971 for nOPV2-c1) presented in aggregate as a function of EES day. **c** Cre5 123/179 in aggregate for nOPV-c1. All variants reported have Q scores ≥ 30.

Variants in domain V and domain IV (U398C in Sabin 2 and nOPV2-c2, or U459C in nOPV2-c1) were not observed in any of the nOPV2-c2 EES. Variants in VP1-143 resulting in amino acid changes from isoleucine to valine, threonine, or asparagine were observed in day 9 and 28 EES from M4a (Fig. 6a) and day 8 and 9 EES from M4 (Fig. 6b) through polymorphisms at nucleotide 2908 or 2909, with a trend of increasing frequency of polymorphisms for later collection days (Fig. 7b). A supplemental analysis of the FASTQ files confirmed these VP1-143 polymorphisms are not co-located on genomes.

EES were identified for five M4 participants (with OPV-vaccination history) who received nOPV2-c2, with all stools and amplified virus evaluable by NGS (Fig. 7 and Supplementary Fig. 2b). EES ranged from days 5 to 7 post-vaccination. No variants were observed in U398C, domain V, VP1-143, or the modified CpG sites in the capsid region.

### Next-generation sequencing assessment of amplified virus

**Fig. 8:**
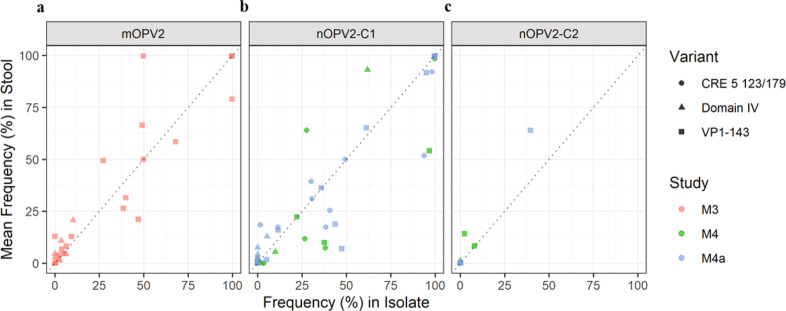
Comparison of average frequency of polymorphisms measured in EES stools and corresponding cell culture isolate for polymorphisms of interest. **a.** mOPV2, **b.** nOPV2-c1 and **c**. nOPV2-c2 . Domain IV represents nucleotide position 398 for mOPV2/nOPV2-c2 and 459 for nOPV2-c1. VP1-143 and cre5 123/179 polymorphisms presented in aggregate for each virus. The dotted diagonal line represents the identity (1:1) line. If variant is only present in 1 stool that value is presented. If variant is not present in either, assigned 0 for plotting purposes. All variants reported have Q scores ≥ 30.

To confirm whether the culture-amplified virus was sufficiently representative for neurovirulence assessment, the frequency of mutation in the stools was compared to the frequency of mutation in the amplified sample for mOPV2, nOPV2-c1 and nOPV2-c2 (Figs. 4–6) with generally similar results. Polymorphism frequencies were reviewed in more detail for the attenuation sites and, for nOPV2-c1, variants in the relocated cre5 (Fig. 8). In this comparison, no polymorphisms were detected in the isolate without also being detected in at least one of the stool replicates. For mOPV2, nOPV2-c1 and nOPV2-c2 high concordance (correlation coefficient 0.928, 0.947 and 0.984 respectively) and little evidence of bias from the amplification process were observed. Accordingly, the amplified virus samples were determined to be suitably representative for neurovirulence testing.

### Neurovirulence of shed virus

**Fig. 9:**
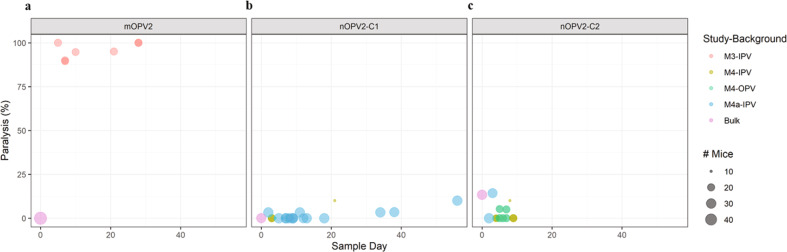
Measured paralysis rate in modified mouse neurovirulence test. Vaccine bulk lots and culture-amplified virus (10^4^ CCID_50_/5 ul dose) from each EES were tested in replicates as indicated by plot size. Paralysis rate per administered vaccine bulk lot is shown on day 0. Paralysis rate for shed virus samples is indicated versus day of EES. **a** mOPV2, **b** nOPV2-c1 **c** nOPV2-c2.

Neurovirulence in mice was measured for the administered mOPV2 lot and culture-amplified EES from participants from the mOPV2 Phase 4 trial (M3). A subset of seven EES were selected for analysis prior to receipt of NGS results, with sub-sampling focusing more heavily on EES collected at earlier time points to attempt to have more diversity in the extent of reversion. All seven EES from individuals who received mOPV2 in the M3 trial showed high levels of paralysis in the mice, with paralysis rates ranging from 89% to 100% (Fig. 9a). Comparisons between paralysis rate and levels of A481G and VP1-143 mutations are shown in Fig. 4 and Supplementary Table 3 with the presence of A481G at high levels associated with high paralysis rates; effect, if any, of VP1-143 variants was not observed. In comparison, the mOPV2 vaccine lot administered also was tested at the same dose over several runs, with 0/50 mice paralyzed.

### Shed nOPV2-c1 virus from phase 1 and 2 trials

From phase 1 (M4a), fifteen EES from nOPV2-c1 recipients were successfully amplified to sufficient titre for mouse neurovirulence testing. From phase 2 (M4) trial, five and four EES were evaluable from the cohorts with IPV-only and OPV vaccination histories, respectively. The data from the three cohorts and the administered vaccine are overlayed in Fig. 9b.

From phase 1 (M4a) study, both the clinical trial lot and the 15 analyzed EES (clinical trial lot and EES each tested in triplicate) showed low to no paralysis, with a mean paralysis rate of 0% (0/29 mice paralyzed) for the administered trial lot and a mean of 1.6% (range of 0–10%) for the EES (Figs. 5a, 9b). Notably, EES virus from day 56, which included nearly fixed variants U123C and U172C in cre5, U459C in domain IV, A2969G coding for VP1-I143V and U4540C (silent polymorphism in cre knockout region) induced paralysis in only 3 out of 30 mice tested. In comparison, a Sabin 2 sample collected day 7 after mOPV2 vaccination with 88% A481G reversion serving as an informal comparator during testing induced paralysis in 90% of mice.

Similar results were seen for shed nOPV2-c1 viruses from phase 2 (M4) study. For the cohort with prior IPV-only exposure, no neurovirulence was associated with EES collected on days 3, 8, and 9. A paralysis rate of 10% was seen from the EES collected on day 21 that contained the U123C polymorphism and reversion in VP1-143. For the cohort with prior OPV exposure, the four evaluable EES were from days 4 to 8 post-vaccination with no paralysis induced by any sample (Supplementary Fig. 2a).

### Shed nOPV2-c2 virus from phase 1 and 2 trials

From phase 1 (M4a) and phase 2 (M4) trials, nOPV2-c2 data were more limited with twelve EES successfully amplified in cell culture for evaluation. These data are summarized in Fig. 9c. From M4a, data were limited to two EES from day 2 and 3 post-vaccination with paralysis rates of 0% (0/30) and 14% (4/28), respectively (Fig. 6a). Concurrent analysis of the administered nOPV2-c2 lot yielded a paralysis rate of 13% (4/30 mice paralyzed). EES collected at later time points (day 9 and 28) showed more reversion in VP1-143 by NGS but were not successfully culture-amplified to sufficient titre for the mTgmNVT. However, the neurovirulence of molecular clones with reversion at this site is reported below.

For phase 2 (M4) study, three and five EES were evaluable from the cohorts with IPV- and OPV-vaccination histories, respectively. In the IPV-background cohort, two EES induced no paralysis, while a third collected on day 8 post-vaccination yielded 10% paralysis (1/10) (Fig. 6b). This sample had VP1-I143T reversion at approximately 15% in the culture-amplified sample. For the cohort with prior OPV exposure, the samples induced either 0% or 5% paralysis rates, with no reversion detected by NGS.

### Neurovirulence of viruses derived through molecular cloning

A series of strains were created using site-directed mutagenesis to incorporate polymorphisms seen in EES to more completely evaluate their impact on neurovirulence, with the calculated 50% paralytic dose levels (PD_50_) summarized in Table 1.

**Table 1:** 50% paralytic dose (PD_50_) concentration (log10 CCID_50_) for various virus strains generated by molecular cloning to contain key mutations identified in the EES.

Strain	PD_50_^a^
Sabin-2	5.9 (5.5–6.2)
Sabin-2/A481G	1.8 (1.4–2.1)
Sabin-2/U398C	4.8 (4.2–5.3)
Sabin-2/U398C/A481G	1.9 (1.4–2.3)
Sabin-2/VP1-I143V	3.5 (2.9–4.0)
Sabin-2/A481G/VP1-I143V	1.2 (0.8–1.6)
Sabin-2/U398C/A481G/VP1-I143V	1.2 (0.9–1.5)
nOPV2-c1	>8.4 (0/8)^b^
nOPV2-c1/123C	>6.5 (2/8)^b^
nOPV2-c1/VP1-I143V	7.8 (7.4–8.3)
nOPV2-c1/U123C/U459C/VP1-I143V	4.3 (3.8–4.7)
nOPV2-c2	6.3 (5.8–6.8)
nOPV2-c2/U398C	4.0 (3.3–4.6)
nOPV2-c2/VP1-I143V	3.9 (3.3–4.6)
nOPV2-c2/ U398C/VP1-I143V	2.4 (1.8–3.0)

^a^Virulence of viruses was determined in Tg66 mice using intraspinal inoculation (see Supplementary Material for more information on Tg66 mice).

^b^Proportion of mice paralysed at highest dose.

For Sabin 2-derived clones, each of the three variants discussed earlier (U398C, A481G, and VP1-143 variants) all increase virulence with A481G having the largest impact.

nOPV2-c1 shows a lower initial virulence than Sabin 2, and nOPV2-c1 variants with VP1-143 reversion or with the cre5 polymorphism (U123C) both remain less neurovirulent than unreverted Sabin 2. Combining these two variants in one nOPV-c1 clone with U459C in domain IV results in a strain that is more virulent (PD_50_ = 4.3 log CCID_50_) than Sabin 2 vaccine, but notably less virulent than Sabin 2 with the A481G reversion alone (PD_50_ = 1.8 log CCID_50_) and Sabin 2 with U398C, A481G and VP1-143 (PD_50_ = 1.2 log CCID_50_).

For nOPV2-c2, the initial neurovirulence is similar to unmodified Sabin 2. The U398C (PD_50_ = 4.0 log CCID_50_) or VP1-I143V variants (PD_50_ = 3.9 log CCID_50_) alone or paired together also remain less virulent than Sabin 2 with the A481G reversion.

## Discussion

In this work, an algorithm was developed which allowed the selection of the latest stool sample from each trial participant which could be reliably culture-amplified for genetic heterogeneity and neurovirulence assessments. The selection criterion for the EES (last stool with titre >4 log_10_ CCID_50_/g) was arrived at empirically (see Fig. 2) but also followed logically from the standard cell-culture inoculum size used for amplifications. Specifically, the amplification process used four 50 microliter infections of 10% stool suspension, which would correspond to approximately 50 CCID_50_ or ~35 infectious units per infection at the 4 log_10_ CCID_50_/g threshold. Additionally, sampling artifacts for non-fixed variants would become prominent using lower titre samples, as Fig. 2 shows. The selection of this specific titre threshold had the additional, fortuitous benefit of allowing the EES to be loosely interpreted as the latest sample containing transmissible virus, consistent with what has been described previously^25,26^.

In children with an IPV-only vaccination history who received mOPV2 (study M3), we confirmed prior observations^5–7^ of rapid and high-level reversion of A481G in domain V, with all 18 EES showing reversion (mean 87% with range of 7–99.7%, see Figs. 4, 7a). Polymorphisms in the codon for VP1-143 were also common, with reversion detected in all but one sample. The accumulation of VP1-143 variants to higher levels generally appeared slower than for A481G, consistent with prior reports^6^.

The neurovirulence of 10^4^ CCID_50_ inocula of culture-amplified mOPV2 EES was uniformly high (≥89%), which was anticipated given that the estimated 50% paralytic dose (PD_50_) for a Sabin 2 molecular clone with A481G is 1.8 log CCID_50_ in a similar strain of transgenic mice (Table 1). Conversely, no paralysis (0/50 mice) was observed upon administering the mOPV2 vaccine at the same dose, which was also anticipated given the estimated PD_50_ of 5.9 log CCID_50_ for a Sabin 2 clone (Table 1). These observations validate the sensitivity of the modified mouse neurovirulence test to detect the early and clinically relevant reversion of mOPV2.

The reversion of the Sabin 2 vaccine at both the primary and secondary attenuation sites, with commensurate increases in neurovirulence contrasted results from the nOPV2 candidates.

NGS of the samples showed promising genetic stability results for both nOPV2 candidates. The modified domain V is stabilized through exclusive use of C–G and U–A genetic pairs in stem regions (instead of U–G pairs). As a result, strengthening of a U–A base pair would require either simultaneous mutation of both positions or sequential A to G and then U to C changes to become a stronger G–C pair. For the shed nOPV2-c1 and nOPV2-c2, no pair-strengthening polymorphisms in domain V were detected. Other polymorphisms which were detected in domain V were generally at low levels and all appear to destabilize base pairs, theoretically further attenuating the strains.

NGS on the EES did identify the accumulation of other polymorphisms in the nOPV2 strains. Both candidates showed reversion in the unprotected secondary attenuation site (VP1-143) over time, as seen with Sabin 2^6^. There were no obvious differences in the accumulation of VP1-143 variants for nOPV2-c1 in comparison to mOPV2; however, fewer reversions were seen for nOPV2-c2, likely due to the EES being from earlier days (Fig. 7). Reversion in domain IV (nucleotide 398 in Sabin-2, which is known to be related to virus fitness) was seen for nOPV2-c1 but not nOPV2-c2, likely for similar reasons as described for VP1-143. The appearance of more domain IV reversion in nOPV2-c1 than mOPV2 is in part due to the cessation of sample collection in the mOPV2 study at day 28 post-vaccination whereas in the M4a study samples were collected daily until cessation of shedding.

Mutations that strengthened a base pair in the cre stem (nucleotides 123 and 179) were consistently selected for nOPV2-c1 (Fig. 7a, structure in Supplementary Fig. 1), suggesting that they confer a fitness advantage in vivo. An nOPV2-c1 molecular clone with U123C showed that this polymorphism also modestly increased neurovirulence (Table 1).

Neurovirulence was determined for candidate bulks and shed nOPV2 viruses, and is informally contrasted with the shed Sabin 2 viruses from the M3 study (Fig. 9). Candidate vaccine bulks and the majority of EES were tested more than once and in separate tests. Assay controls (Sabin 2 SO + 2/II) met preset acceptance criteria for all tests and for the 33 EES tested in triplicate or duplicate by the mTgmNVT, it was determined that 88% (29/33) of tests from the various trials and vaccines were within an 11% (1 mouse) range for all replicates, indicating that the assay is consistent and reliable.

Limited increases in virulence were seen for shed nOPV2-c1, with measured paralysis rates ranging from 0% to 10%. The day 56 EES (M4a trial) showed 10% paralysis (3/30 mice paralyzed) and had fixed mutations at cre, position 459 in domain IV and at VP1-143. The relatively low virulence of late-shed viruses that have fixed mutations at these three sites is a promising result and is consistent with studies using molecular clones (Table 1)^27^. It suggests that any significant further increases in virulence would require mechanism(s) of reversion not observed in these clinical trials.

More limited results were available for nOPV2-c2, mainly because fewer EES were identified due to reduced shedding^17,19^, suggesting this candidate may have a shorter period of transmissibility. The available sequencing results are similar to nOPV2-c1 in that reverting mutations in the stabilized domain V do not seem to be present, while reversion at the secondary attenuation site, VP1-143, is observed. As for nOPV2-c1, the overall impression is that nOPV2-c2 is also considerably less likely than Sabin-2 to evolve towards significant cVDPV2 virulence.

Because of the safety, immunogenicity and promising phenotypic stability of these viruses, nOPV2 use under Emergency Use Listing (EUL) features prominently in the Global Polio Eradication Initiatives new strategy to stop further spread of cVDPV2^1^, and the EUL has recently been granted for nOPV2-c1. The methods described here are now being applied to shed virus from paired phase 4 (NCT02521974) and phase 2 (NCT03554798) clinical trials of mOPV2 and nOPV2 in children and infants, allowing more direct comparison of the molecular evolution and virulence of shed nOPV2 viruses with shed Sabin 2 in the age groups that will be the focus of outbreak responses.

## Methods

### Sabin 2 samples from mOPV2 clinical trial used for development work

De-identified fecal samples from 4 infants in a clinical trial involving mOPV2 (Polio Sabin Mono Two, GlaxoSmithKline Biologicals, Belgium) challenge at 18 or 40 weeks following a mixed bOPV-IPV vaccination schedule^22^ were used for assay development. The protocol was approved by ethics committees and as appropriate by National Regulatory Authorities, the Colorado Multiple Institutional Review Board, and the Western Institutional Review Board. Parents or guardians of all infants supplied written informed consent before enrolment. Stool samples were collected before and on days 7, 14, 21 and 28 post-mOPV2 challenge and shipped to CDC. Type 2 poliovirus was detected by RT-PCR^28^ and PCR-positive samples titrated in culture as CCID_50_ by a modification of the WHO cell sensitivity assay^29^. Samples for development work were selected for a range of viral titres in stool.

### Phase 4 clinical trial with mOPV2

A phase 4 trial (M3 study, NCT02582255) was conducted in which mOPV2 (Polio Sabin Mono Two, GlaxoSmithKline Biologicals, Belgium) was administered to 100 healthy 1- to 5-year-old Lithuanian children who had previously received at least three IPV vaccinations^30^. The protocol was approved by the local institutional review board. Written consent was obtained from 2 parents or legal guardian(s) at enrolment per country regulations. Trial participants were randomized to receive either one or two doses of mOPV2, with the second dose occurring 4 weeks after the first. Stool samples were collected daily until day 10 following each vaccination, and then on days 14, 21, and 28. Type 2 poliovirus was detected by RT-PCR^28^ and PCR-positive samples titrated in culture as CCID_50_ per gram of stool^29^. For each participant, the last collected sample with at least 4.00 log_10_ CCID_50_ per gram of stool was termed the EES, with subsequent analysis as described.

### Novel OPV2 candidates

The nOPV2 vaccine candidates are attenuated serotype 2 polioviruses derived from a modified Sabin type-2 infectious cDNA clone. The two candidates (nOPV2-c1, nOPV2-c2) both include the previously described S15 genetic stabilization of domain V in the 5′ untranslated region^11^. Briefly, nOPV2-c1 also includes relocation and modification of the cis-acting replication element (cre) to the 5′ end of the genome, inactivation of cre element by introducing silent mutations, and modifications of the 3D polymerase protein to increase fidelity and reduce recombination^27^. In addition to the domain V modification, nOPV2-c2 includes synonymous codon deoptimization of the capsid (P1) region, with approximately 40% of the available sites modified through incorporation of CpG dinucleotides in the second and third positions of the codons^15^.

Research stocks of the strains, and similar strains (molecular clones) used for characterization studies, were recovered through standard methods of RNA transcription, transfection and amplification as previously described^31,32^. Production lots of both nOPV2 candidates were manufactured and released by P.T. Bio Farma (Bandung, Indonesia).

### Samples from nOPV2 phase 1 clinical trial

Phase 1 study (M4a, NCT03430349) in a containment facility^18^ was conducted with the two nOPV2 vaccine candidates^17^. Healthy, adult volunteers (18 to 50 years) with exclusive IPV polio vaccination history were enrolled sequentially in two groups of 15 participants, each group receiving one of the two vaccine candidates at a dose of approximately 10^6^ CCID_50_. Ethical approval was received from university and hospital institutional review boards for the study and all participants provided written consent at enrolment.

Daily stool samples were collected until cessation of virus shed and periodically shipped to the Centers for Disease Control and Prevention (CDC, Atlanta, GA, USA), where they were stored at −20 °C until analysis. Type-2 poliovirus genomes were detected using a Sabin multiplex real-time RT-PCR (rRT-PCR) assay of total nucleic acid extracted from stool suspensions (50%, w/v)^33^. In samples positive for type-2 poliovirus, infectious virus was measured as CCID_50_/g stool as described above^29^.

Stool suspensions (10% w/v in EMEM, Gibco) were prepared for next-generation sequencing (NGS). To achieve the required concentration of virus for mouse neurovirulence assays, virus was amplified in HEp-2C (ATCC, CCL-23) cells maintained in EMEM supplemented with 2% fetal bovine serum (Atlanta Biologicals). Confluent monolayers of HEp-2C cells in 24-well cell-culture cluster plates (Costar) were inoculated with a 50-µL aliquot of EES suspension (4 wells, at least 50 CCID_50_ per well) and incubated for 3 days at 33 °C, 5% CO_2_. Following two freeze-thaw cycles, cell lysates from the 4 wells were pooled, and virus isolates were harvested following removal of cell debris by centrifugation at 3000 x *g* for 10 min. The stool suspensions and virus isolates were assigned unique identifiers and shipped on dry ice to Viroclinics Biosciences B.V. (Rotterdam, the Netherlands) and stored frozen at −20 °C until analysis.

### Samples from nOPV2 phase 2 clinical trial

In the M4 phase 2 trial (EudraCT: 2018-001684-22), 117 or 116 healthy adult (18–50-year old) participants were each administered orally at least one dose (approximately 10^6^ CCID_50_) of nOPV2-c1 or nOPV2-c2. For each candidate approximately 100 participants had prior trivalent oral poliovirus (tOPV) immunization. A subset of 17 (nOPV2-c1) or 16 (nOPV2-c2) participants had prior IPV immunization exclusively. Study protocols were approved by each centre’s institutional review board and the Belgian national authority. All participants provided written consent at enrolment.

Stool samples were collected post initial dose daily on days 0 to 10, then on days 14, 21 and 28 following vaccine candidate administration and shipped to CDC (Atlanta, GA, USA). Poliovirus genomes were detected and titrated as described above to identify EES for analysis by NGS and the modified mouse neurovirulence test.

### Neurovirulence of shed virus

The WHO poliovirus receptor (PVR) transgenic mouse neurovirulence test (TgmNVT) used to release vaccine^24^ was modified (*m*TgmNVT, Supplementary Table 1) to assess the neurovirulence of shed virus from clinical trials. The titres of Sabin OPV2 WHO international standard 15/296 (NIBSC) and virus isolates (from EES) were determined by CCID_50_ assay^29^ in Hep-2C cells at Viroclinics Biosciences B.V. prior to conduct of the mTgmNVT. For each EES, ten 6–8-week-old Tg-PVR21 mice (CLEA/Japan) (5 of each sex) received intraspinal inoculations of 10^4^ CCID_50_ amplified virus in 5 µl. Each EES from the M4a phase 1 trial was tested in triplicate. EES from the M3 trial were tested in duplicate and from the M4 phase 2 trial at least once. Clinical trial bulk preparations of both vaccine candidates were also tested at the 10^4^ CCID_50_ dose. The underlying methodology leading to the selection of the 10^4^ CCID_50_ dose is summarized in Fig. 3. Controls included 20 mice inoculated with Sabin OPV2 virus SO + 2/II at the 5.0 and 6.0 log_10_ CCID_50_ dose levels, as well as a sample of shed Sabin 2 virus collected Day 7 post-mOPV2-challenge in a prior clinical trial^22^. Back-titrations of the diluted samples confirmed that titres were within 0.5 log CCID_50_ of the nominal dose. Inoculated mice were monitored for paralysis over a 14-day period per established protocol^24^.

In some cases, the paralytic dose for 50% of mice (PD_50_) was determined. For these tests, groups of 10 Tg-PVR21 mice (or, where indicated, 8 of Tg66 mice, laboratory research line expressing PVR developed at NIBSC) were inoculated with each dose and PD_50_ calculated using the Spearman-Kärber method.

The Tg-PVR21 mouse experiments at Viroclinics were conducted in compliance with Dutch Animal Testing Act (WOD) and according to the DIRECTIVE 2010/63/EU of The European Parliament and of the Council of 22 September 2010 on the protection of animals used for scientific purposes under project license 2770020171404 issued by the central authorizing body (Centrale Commissie Dierproven). Ethical approval for the study was obtained in working protocols 1-3 from the animal ethics committee (Instantie voor Dierenwelzijn) under the above project license.

The Tg66 mouse experiments at NIBSC were performed under licenses PPL 80/2478 and PPL 70/8979 granted by the UK Home Office under the Animal (Scientific Procedures) Act 1986 revised 2013 and reviewed by the internal NIBSC Animal Welfare and Ethics Review Board.

### Next-generation sequencing (NGS) of shed virus

NGS and data analysis were performed on viral RNA isolated from both cell-culture-amplified virus and from 10% stool suspensions of the EES of each participant, using a previously described method^20,21^. The method was qualified and executed at Viroclinics Biosciences B.V. In brief, viral RNA was isolated from 140 µl amplified virus stock or stool suspension of each EES using QIAmp Viral RNA mini kit (Qiagen). cDNA preparation and amplification of full-length genome were performed as described elsewhere^7^. Tagmentation and library preparation by Nextera XT (Illumina) were performed, followed by 300-cycle paired-end sequencing using MiSeq reagent kit v3 reagents on a MiSeq instrument with analysis software version 1.8.46 (Illumina) to generate FASTQ files. Data analysis was performed using the relevant candidate reference sequence (or to Sabin 2 reference sequence; Genbank accession number AY184220) and a proprietary algorithm at Viroclinics comprised of trimming (Trimgalore version 0.4.4), mapping (BWA mem; version 0.7.16a-r1181), and SNP (single nucleotide polymorphism) variant calling where multinucleotide polymorphisms and COMPLEXES are split into multiple SNPs using the vcfallelicprimitives utility from vcflib library (freebayes; version v1.1.0-60-gc15b070). Polymorphisms (present at ≥1%) were reported as single nucleotide polymorphisms (SNPs) with the impact on amino acid coding of the SNP noted per SNP. Re-analysis of select FASTQ files was conducted in Geneious Prime to assess the possible co-location of SNPs in the VP1-143 codon.

The criteria for reporting in Supplementary Tables 3–12 are described below. Data provided in these Tables are represented in Figs. 4–6 utilizing a colour gradient scale. The mean frequency of a variant of both stool replicates is represented along with the frequency of the variant observed in the culture-amplified replicate for each EES. Where relevant, the figures also show the corresponding mTgmNVT result for each EES.

#### Reporting variants in known attenuation sites and modified regions of shed viruses

Certain EES only had one or two evaluable components (replicates). For EES where both stool aliquots and culture-amplified virus isolate were evaluable, variants in known attenuation sites and modified regions of the strain present in at least two of the three replicates are indicated in Supplementary Tables 3–12. Similarly, if only two replicates of a sample (two stool or one stool and isolate) were available, and a variant was present in both replicates, the variant is indicated in the results tables. If a variant was present in only 1 of 1, 1 of 2, or 1 of 3 available replicates (and thus not confirmed), it was only indicated in tables if present at ≥10% frequency. However, once a reporting threshold for a variant was met, all positive results for other EES derived from that vaccine/candidate were tabulated in results for that study, even if only present in a single replicate.

All variants reported have Quality scores (Q scores) ≥ 30 unless otherwise indicated in the supplementary tables.

#### Reporting variants in other regions of shed viruses

Variants associated with amino acid changes present in other regions (outside of key attenuating and modified regions noted above) and the CpG modified region of nOPV2-c2 are provided in supplementary Tables 3–12. If the mean frequency of a variant associated with an amino acid change was 50% for an EES (regardless of the number of replicates available per EES), the variant was tabulated. In cases where EES meet this criterion for a particular variant, other EES showing the variant were also tabulated even if present below 50%. If a fixed variant (≥95%) associated with an amino acid change was observed in at least one of the replicates it was also tabulated.

### Reporting summary

Further information on research design is available in the Nature Research Reporting Summary linked to this article.

## Supplementary information

Supplementary Information

Reporting Summary

## Data Availability

Datasets specific to this publication are available from the corresponding author upon reasonable request and contingent on the requested uses being permitted under the informed consent received from the source clinical study, where applicable. The full genome sequence of nOPV2 candidate 1 can be found in GenBank (accession number MZ245455). The full genome sequence of nOPV2 candidate 2 can be found in GenBank (accession number MN654096). Sequencing data can be accessed on the SRA database (accession number PRJNA735689).

## Code availability

Algorithms for variant selection are described under METHODS. R codes for tables and figures specific to this publication are available from the corresponding author upon reasonable request.
