# Supplementary material for: Assessment of genetic changes and neurovirulence of shed Sabin and novel type 2 oral polio vaccine viruses
Source: NPJ Vaccines. 2021 Jul 29;6:94. doi: 10.1038/s41541-021-00355-y (PMC8322168; doi:10.1038/s41541-021-00355-y)
Supplement: Supplementary file 1 — Supplementary Information [file 41541_2021_355_MOESM1_ESM.pdf]

Supplementary Material

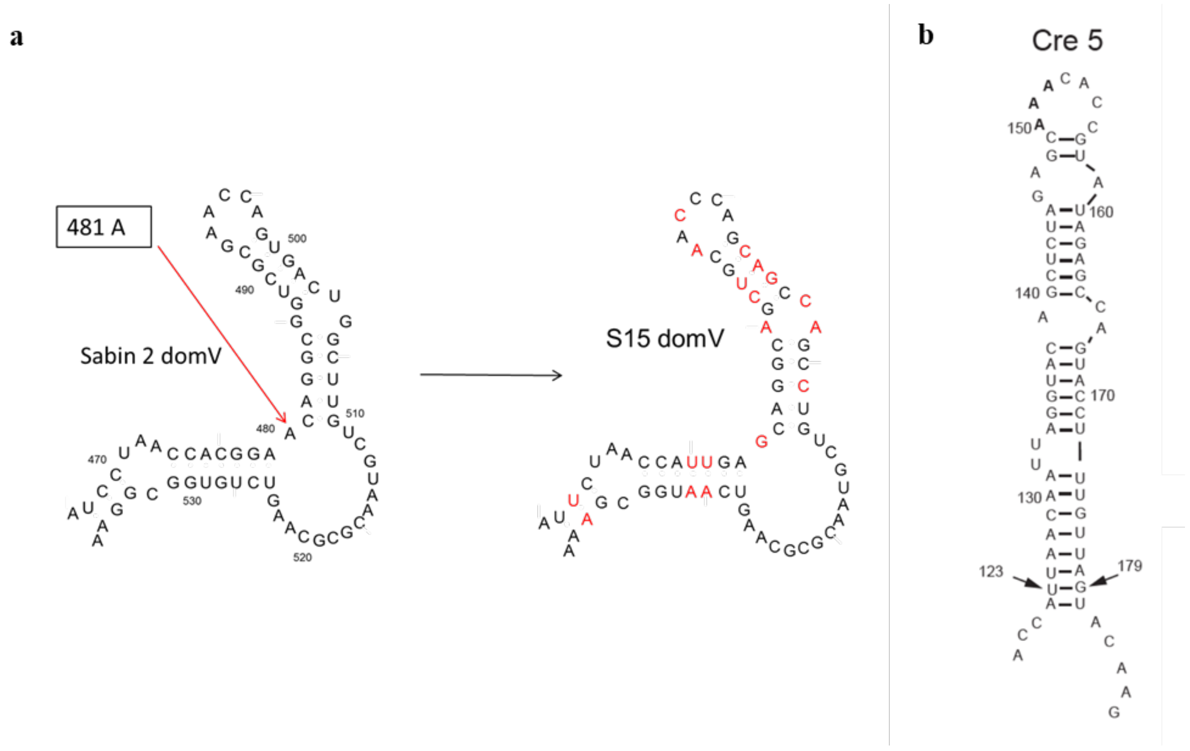

Supplemental Figure 1. Predicted RNA secondary structures. **a.** Representation of Domain V in Sabin-2 and changes in bases (indicated in red) leading to the modified S15 domain V present in both nOPV2c1 and c2. **b.** Structure of relocated cre5 present in nOPV2-c1.

Supplemental Table 1. Comparison of WHO vaccine release test and modified mouse neurovirulence test used to test shed virus in clinical samples

	WHO TgmNVT	Modified TgmNVT
Purpose	Vaccine release	Neurovirulence of virus shed in stool
Mouse type/age[1]	Tg PVR21 (6-8 wks old)	Tg PVR21 (6-8 wks old)
Sample type	Monovalent vaccine bulk	Virus amplified from stool[2]
Dose level administered to mice	2 doses (6 and 5 $\log_{10}$ $CCID_{50}$/5ul)	1 dose (4 $\log_{10}$ $CCID_{50}$/5ul)
Route of administration	Intraspinal	Intraspinal
Number of mice	32 per dose, 16 per gender (64 per vaccine and reference)	10 per stool sample analyzed, 5 per gender and 20 per reference dose with 10 per gender
Duration	14 days clinical observation	14 days clinical observation
Clinical scoring	Per criteria in section 5.2 of WHO SOP	Per criteria in section 5.2 of WHO SOP
Reference	WHO Sabin 2 reference virus (SO+2/II)	WHO Sabin 2 reference virus (SO+2/II)
Test validity	Test invalid if:<br>• SO+2/II 6 log10 dose >95% paralysis<br>• SO+2/II 5 log10 dose <5% paralysis<br>• >5% mice excluded	Test invalid if:<br>• SO+2/II 6 log10 dose ≤ 15% paralysis<br>• SO+2/II 5 log10 dose ≥ 35% paralysis<br>• >20% SO+2/II mice excluded
Analysis	Logistic regression, comparison via log odds ratio of the proportion of mice paralyzed at each dose of the reference and test vaccines – pass/fail	Logistic regression, comparison via odds ratio of the proportion of mice paralyzed in nOPV2 test samples relative to test samples from mOPV2 recipients, independent of reference used to monitor assay integrity.
Personnel	Inoculators and clinical scorers trained per Appendix 2 of WHO SOP	Inoculators and clinical scorers trained per Appendix 2 of WHO SOP

[1]*Mice shipped at 5wks of age*
[2]*From a single sample per participant*

Supplemental Table 2. Correlation of frequency of polymorphisms in virus in stool versus cell culture amplified virus for eight fecal samples with shed Sabin-2

**Stool titre, $CCID_{50}$/g**	**Culture amplified titre, $CCID_{50}$/ml**	**Correlation coefficient**
7.97	9.50	0.9801
7.38	9.50	0.9416
6.25	9.50	0.9810
4.47	9.50	0.8499
4.44	8.25	0.7231
3.41	2.90	0.3996
3.31	1.70	0.5131
2.84	9.50	0.1865

Supplemental Table 3. *M3 mOPV2.* Frequency of variants in EES at known attenuation sites

EES_day (Samples)	DomIV	DomV					VP1-143					% Paralysis (mice paralyzed/total mice)
	**T398C**	**T471C**	**A480G**	**A481G**	**G493A**	**T500C**	**A2908T I143F**	**A2908G I143V**	**T2909C I143T**	**T2909A I143N**	**T2909G I143S**	
5 (SSI)	0.03,0.02,0		0.01*,0,0	0.89,0.83,0.89	0.02,0,0		0.1,0.18,0.17	0,0,0.01	0.07,0.03,0.08	0.02,0,0.03	0,0,0.03	100.0 (20/20)
6 (SSI)	0.01,0,0.03			0.55,0.79,0.62	0.18,0,0.11	0.04,0.13,0.06		0.05,0.13,0.07	0,0,0.02		0.02,0,0.03	
7 (SSI)			0.01*,0,0.01*	0.83,0.8,0.87					0.21,0.21,0.47			89.5 (17/19)
7 (SSI)			0.01*,0,0	0.79,0.8,0.77				0.02,0.01,0	0.07,0.08,0.05			90.0 (18/20)
10 (SSI)			0.01*,0,0.01*	0.91,0.9,0.92	0.01,0,0			0.07,0.04,0	0.43,0.51,0.39			94.7 (18/19)
10 (SSI)	0.04,0,0.06		0,0.01*,0	0.97,1,0.97				0.03,0.09,0.03	0.2,0.26,0.23	0.07,0,0.01		
10 (SSI)	0.16,0.25,0.1			1,1,0.98					0.68,0.49,0.68			
14 (SSI)				0.98,1,0.98				1,0,0	0,0.58,1			
14 (SSI)	0.04,0,0	0.04,0,0.06		0.96,1,0.96	0.03,0,0	0.01,0,0		0.21,1,0.24	0.32,0,0.56	0.02,0,0	0.02,0,0	
14 (SSI)	0.02,0.03,0.02		0,0,0.01*	0.68,0.6,0.74				0,0.06,0.02	0,0.04,0	0.02,0,0		
21 (SSI)		0,0,0.01		0.98,1,0.98				0.41,0.38,0.28	0.59,0.62,0.72			95.0 (19/20)
21 (SSI)				0.98,0.97,0.97				0.2,0,0	0,0.05,0			
21 (SSI)				0.09,0.04,0.1		0,0.01,0						
21 (SSI)				1,1,0.98				0,1,0.46	1,0,0.53			
26 (SSI)	0,0.11,0.03		0,0.01*,0	1,1,0.98				0.04,0,0.03	0.71,0.74,0.52			
28 (SSI)				0.98,1,0.98				0.07,0,0.03	0.93,1,0.95			100.0 (20/20)
28 (SI)			0.01*,0	1,0.98					1,1			100.0 (20/20)
29 (SSI)			0.01*,0,0	1,1,0.98					1,1,1			

EES day shown with stool 1, stool 2 and cell culture isolate (SSI), if present. Variant and associated amino acid change indicated, if applicable. Blank cells = variant not detected in stool or isolate. NGS pipeline reports the variants as SNPs. Coding impact assumes changes are not in common genomes when multiple variants are observed in VP1-143 within the same sample. Paralysis rates are indicated for the samples that have mTgmNVT results available. Grey cells = mTgmNVT result not available.

*Q<30 for variant in this replicate.

Supplemental Table 4. *M3 mOPV2.* Frequency of variants associated with amino acid changes in other regions of mOPV2

EES_ day (Samples)	VP4		VP2				VP3			VP1		2B	2C	CRE	2C	3A		% Paralysis (mice paralyzed/total mice)
	T763A S6T	T766A S7T	G1068T W38C	C1168T R72C	G1426A G158R	C1463T A170V	A1997G H77R	C2000A S78Y	A2074G I103V	C2546T T22I	A2992G N171D	A4093G I88V	A4441G T107A	G4456A V112I	C4577T T152I	TAAAAAT5132TAAAT 10I>^	C5156T P16L	
5 (SSI)			0.01,0,0															100.0 (20/20)
6 (SSI)									0.03,0.19,0.04									
7 (SSI)							0,0.02,0											89.5 (17/19)
7 (SSI)											0,0.01,0							90.0 (18/20)
10 (SSI)							0.06,0.01,0.04				0.07,0.06,0.02							94.7 (18/19)
10 (SSI)									0.02,0.02,0.04									
10 (SSI)										0.01,0,0.01	0,0.04,0							
14 (SSI)		1,0,0									0,0.4,0							
14 (SSI)				0,0,0.02					0.09,0,0.12		0.07,0,0			0.02,0,0				
14 (SSI)	1,1,0.99																	
21 (SSI)						0,0,0.02		0.92,0.94,0.88						0.58,0.61,0.7				95.0 (19/20)
21 (SSI)							0.26,0.47,0.92		0.58,0.7,0.83		0,0.05,0	0,0.04,0						
21 (SSI)			0.02,0,0			0,0,0.02			0,0,0.01				0.02,0,0					

EES_ day (Samples)	VP4		VP2				VP3			VP1		2B	2C	CRE	2C	3A		% Paralysis (mice paralyzed/total mice)
	T763A S6T	T766A S7T	G1068T W38C	C1168T R72C	G1426A G158R	C1463T A170V	A1997G H77R	C2000A S78Y	A2074G I103V	C2546T T22I	A2992G N171D	A4093G I88V	A4441G T107A	G4456A V112I	C4577T T152I	TAAAAAT5132TAAAT 10I>^	C5156T P16L	
21 (SSI)				0,1,0.44						0,1,0.45								
26 (SSI)				0,0.14,0.05			0.84, 0.76, 0.9				0,0.04, 0			0,0.04,0				
28 (SSI)							0.25, 1,0.14				0.32,1, 0.36		0,1,0		0,0.99, 0		0,0.99, 0	100.0 (20/20)
28 (SI)				0.07,0.01	0.88,0.93	0.92, 0.98	0.9,0.97											100.0 (20/20)
29 (SSI)			0,1,0									0,1,0				0,0.99,0		

EES day shown with stool 1, stool 2 and cell culture isolate (SSI), if present. Variant and associated amino acid change indicated, if applicable. ^Amino acid changed undefined. Blank cells = variant not detected in stool or isolate. *Q<30 for variant in this replicate**.** Paralysis rates are indicated for the samples that have mTgmNVT results available. Grey cells = mTgmNVT result not available.

Supplemental Table 5. *M4a nOPV2-c1.* Frequency of variants in EES at known attenuation sites

EES_day (Samples)	CRE5						DomIV	S15 DomV		VP1-143			2C cre KO					3D-pol-38	3D-pol-53	% Paralysis (mice paralyzed/total mice)
	C121T	T123C	A138G	T172C	T172A	G179A	T459C	A541G	C569T	A2969G I143V	T2970C I143T	T2970A I143N	G4517A V112I	C4520A Q113K	T4540C	A4541G I120V	C4543T	A6160G	T6205C	
2 (SSI)																				3.3 (1/30)
5 (SSI)																				0.0 (0/30)
7 (SSI)		0.27,0.3,0.16		0,0,0.03		0.05,0.08,0.07			0,0.12,0.07				0.29,0.11,0.1				0.03,0.01,0			0.0 (0/30)
7 (SSI)										0.93,0.9,0.95										0.0 (0/30)
8 (SSI)		0.22,0.15,0.02					0.09,0.17,0.05			0.02,0.01,0	0.18,0.12,0.47									0.0 (0/30)
9 (SSI)		0.94,0.9,0.98	0,0,0.14							0.44,0.38,0.84	0.05,0.06,0.03							0.01,0.02,0		0.0 (0/29)
9 (SSI)	0,0.11,0.01	0.31,0.03,0.38			0,0.07,0.02		0,0.07,0				1,1,1		0,0.04,0							0.0 (0/30)
9 (SSI)		0.55,0.57,0.58				0.03,0.09,0.03	0.04,0,0			0.02,0,0										0.0 (0/30)
11 (SSI)		0.3,0.28,0.13				0.2,0.23,0.68				0.02,0,0.05	0.02,0,0									3.3 (1/30)
12 (SSI)		0.76,0.77,0.94				0,0.02,0	0.02,0.02,0			0.02,0.03,0	0.74,0.67,0.36									0.0 (0/28)
13 (SSI)	0,0.02,0	0.21,0.33,0.12	0,0.01,0			0.62,0.42,0.49	0.03,0.02,0			0.21,0.14,0.09	0.16,0.16,0.15	0.12,0.16,0.1								0.0 (0/30)
18 (SSI)		1,1,1									0.6,0.71,0.61					0.02,0,0				0.0 (0/30)

EES_day (Samples)	CRE5						DomIV	S15 DomV		VP1-143			2C cre KO					3D-pol-38	3D-pol-53	% Paralysis (mice paralyzed/total mice)
	C121T	T123C	A138G	T172C	T172A	G179A	T459C	A541G	C569T	A2969G I143V	T2970C I143T	T2970A I143N	G4517A V112I	C4520A Q113K	T4540C	A4541G I120V	C4543T	A6160G	T6205C	
34 (SSI)		0.86,0.88,0.88				0.13,0.13,0.1	0.99,0.99,0.99													3.3 (1/30)
38 (SSI)						1,1,1		0.01*,0.01*,0.01*			1,1,1			0,0.01*,0.01*						3.4 (1/29)
56 (SSI)		1,1,1		1,1,1			0.99,0.99,0.99	0.01*,0.01*,0.01*		0.99,0.99,1				0.01*,0.01*,0	0.99,0.99,0.99				0,0.19,0	10.0 (3/30)

EES day shown with stool 1, stool 2 and cell culture isolate (SSI), if present. Variant and associated amino acid change indicated, if applicable. Blank cells = variant not detected in stool or isolate. *Q<30 for variant in this replicate**.** NGS pipeline reports the variants as SNPs. Coding impact assumes changes are not in common genomes when multiple variants are observed in VP1-143 within the same sample. Paralysis rates are indicated for the samples that have mTgmNVT results available. Grey cells = mTgmNVT result not available.

Supplemental Table 6. *M4a nOPV2-c1.* Frequency of variants associated with amino acid changes in other regions of candidate

EES_day (Samples)	VP4			VP2	VP3		VP1						3C-pol	% Paralysis (mice paralyzed/ total mice)
	**C930T A41V**	**C948T A47V**	**T993C I62T**	**G1478A E155K**	**A1987G I53M**	**C2059A H77Q**	**A2582G T14A**	**A2639G S33G**	**C2694T A51V**	**A3053G N171D**	**C3195T A218V**	**G3425A E295K**	**C5694T A66V**	
2 (SSI)	0.13,0.24, 0.25							0.16,0.16,0.18	0.03,0.01, 0.01	0,0.01,0.02		0.03,0.03, 0.07		3.3 (1/30)
5 (SSI)			0.03,0,0.15					0.01*,0,0			0.65,0.5,0.42		0.03,0.06, 0.17	0.0 (0/30)
7 (SSI)	0.01,0,0							0.82,0.83,0.66				0.01,0,0		0.0 (0/30)
7 (SSI)	0.98,0.99, 0.99							0.03*,0.01*, 0.01*				0.02,0,0		0.0 (0/30)
8 (SSI)				0,0.02,0			0.01,0,0	0.01*,0.01*, 0						0.0 (0/30)
9 (SSI)														0.0 (0/29)
9 (SSI)					0.99,0.99, 0.99			0.01*,0.01*, 0				0.97,1,0.99		0.0 (0/30)
9 (SSI)	0.02,0,0	0.06,0.11, 0.06						0.91,0.89,0.94						0.0 (0/30)
11 (SSI)	0.02,0.02, 0							0.01*,0.02*, 0	0.68,0.72, 0.31					3.3 (1/30)
12 (SSI)								0.02*,0.03*, 0						0.0 (0/28)
13 (SSI)	0.42,0.55, 0.62							0.05*,0.04*, 0.14	0,0,0.01					0.0 (0/30)
18 (SSI)				0.6,0.47, 0.83				0.01*,0.01*, 0	0.02,0,0		0.35,0.51, 0.17			0.0 (0/30)
34 (SSI)		0.98,0.99, 1						0.01*,0.01*, 0						3.3 (1/30)
38 (SSI)						0.99,0.98, 0.98	0.99,0.96, 0.97	0.02*,0.02*, 0.03*		0.99,0.99, 0.99				3.4 (1/29)
56 (SSI)			0.99,0.99,1					0.02*,0.02*, 0.02*					0.98,0.98, 0.99	10.0 (3/30)

EES day shown with stool 1, stool 2 and cell culture isolate (SSI), if present. Variant and associated amino acid change indicated, if applicable. Blank cells = variant not detected in stool or isolate. *Q<30 for variant in this replicate**.** Paralysis rates are indicated for the samples that have mTgmNVT results available.

Supplemental Table 7. *M4 nOPV2-c1.* Frequency of variants in EES at known attenuation sites

EES_day (Samples)	Participant vaccination background	CRE5		DomIV	S15 DomV	VP1-143			2C cre KO		% Paralysis (mice paralyzed/total mice)
		T123C	G179A	T459C	C547T	A2969G I143V	T2970G I143S	T2970C I143T	C4519T	T4540C	
3 (SSI)	IPV								0,0.03,0.02		0 (0/20)
4 (SI)	OPV										0 (0/10)
6 (SSI)	OPV									0,0,0.01	0 (0/10)
6 (SSI)	OPV										0 (0/10)
6 (SI)	IPV										
7 (SSI)	OPV					0.56,0.52,0.97					
7 (SSI)	OPV									0.02,0.03,0	
8 (I)	IPV					0.04					0 (0/20)
8 (SSI)	OPV			0,0,0.02							0 (0/10)
8 (SSI)	IPV		1,0.97,1								0 (0/10)
9 (SS)	OPV										
9 (SI)	IPV		0,0.03								0 (0/10)
9 (SSI)	IPV	0.19,0.05,0.46	0.12,0,0.07	0.05,0,0.1	0.01,0,0						
10 (S)	OPV										
10 (SS)	OPV				0.21,0						
21 (SSI)	IPV	0.95,0.96,0.52	0.01,0,0.03	0,0.01,0		0.53,0.52,0.56	0.09,0.12,0.03	0.03,0.04,0.07			10 (1/10)
21 (SI)	IPV		0.07,0.38	0.93,0.62				0.1,0.38			

EES day shown with stool 1, stool 2 and cell culture isolate (SSI), if present. Variant and associated amino acid change indicated, if applicable. Blank cells = variant not detected in stool or isolate. *Q<30 for variant in this replicate**.** NGS pipeline reports the variants as SNPs. Coding impact assumes changes are not in common genomes when multiple variants are observed in VP1-143 within the same sample. Paralysis rates are indicated for the samples that have mTgmNVT results available. Grey cells = mTgmNVT result not available.

Supplemental Table 8. *M4 nOPV2-c1.* Frequency of variants associated with amino acid changes in other regions of candidate

EES_day (Samples)	Participant vaccination background	VP4 C930T A41V	VP2 A1518G N168S	VP3 T1883C Y19H	VP3 G2024A D66N	VP3 T2030C Y68H	VP1 G2850A R103K	VP1 A3047G K169E	VP1 G3425A E295K	VP1 AT3433A L298^	2A G3467A A8T	2A C3597T S51L	3A C5426T H86Y	% Paralysis (mice paralyzed/ total mice)
3 (SSI)	IPV	0.25,0.13,0.24										0.12,0.04,0.04		0 (0/20)
4 (SI)	OPV	0.17,0		0.12,0.03					0,0.54			0.21,0.3		0 (0/10)
6 (SSI)	OPV	0.38,0.57,0.86												0 (0/10)
6 (SSI)	OPV	1,1,0.72												0 (0/10)
6 (SI)	IPV		1,0.69						0,0.04					
7 (SSI)	OPV							1,1,1	0.98,1,1					
7 (SSI)	OPV	0.06,0,0												
8 (I)	IPV											0.57		0 (0/20)
8 (SSI)	OPV						0.69,0.75,0.95							0 (0/10)
8 (SSI)	IPV												1,0,0	0 (0/10)
9 (SS)	OPV	0,0.99												
9 (SI)	IPV	1,1		1,0		1,0				1,0				0 (0/10)
9 (SSI)	IPV	0.43,0.71,0.76												
10 (S)	OPV													
10 (SS)	OPV										0.88,0.52			
21 (SSI)	IPV	0,0,0.03			0.18,0.14,0.05									10 (1/10)
21 (SI)	IPV				0.89,0.62									

EES day shown with stool 1, stool 2 and cell culture isolate (SSI), if present. Variant and associated amino acid change indicated, if applicable. ^Amino acid changed undefined. Blank cells = variant not detected in stool or isolate. *Q<30 for variant in this replicate**.** Paralysis rates are indicated for the samples that have mTgmNVT results available. Grey cells = mTgmNVT result not available.

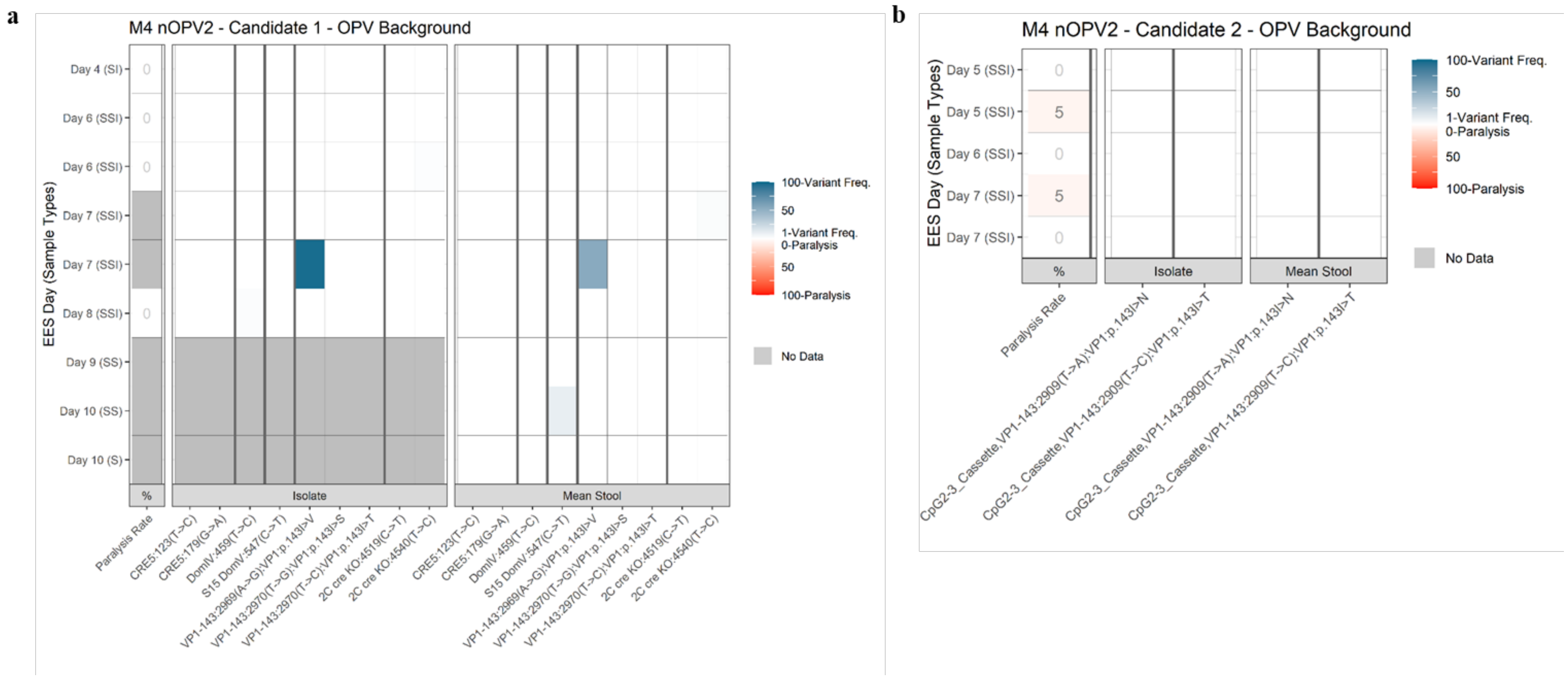

Supplemental Figure 2. *Participant vaccination history is OPV only*. Frequency of variants in EES at known attenuation sites and mTgmNVT results. EES day shown with mTgmNVT result (red colour gradient) and variant frequency (blue colour gradient averaged across two stool replicates (SS) and cell culture amplified virus (I), if present. Amino acid associated with variant indicated, if applicable. White cells = variant not detected. Grey cells = mTgmNVT result not available or no NGS data from stool (S)/cell culture amplified virus (I). **a.** M4 phase 4 trial nOPV2-c1 cohort. **b**. M4 phase 2 trial nOPV2-c2.

Supplemental Table 9. *M4a nOPV2-c2.* Frequency of variants in EES at known attenuation sites

EES_day (Samples)	S15domV			CpG2-3_Cassette, VP1-143		% Paralysis (mice paralyzed/total mice)
	**T478G**	**G479T**	**A480G**	**A2908G I143V**	**T2909C I143T**	
2 (SSI)	0.01*,0.01*,0.01*	0,0.01*,0	0.02*,0.02*,0.02*			0.0 (0/30)
3 (SSI)	0.01*,0.01*,0.01*	0.01*,0.01*,0	0.02*,0.02*,0.02*			14.3 (4/28)
4 (SS)			0,0.01*			
8 (SSI)	0,0,0.01*		0,0.01*,0.01*			
9 (SSI)	0,0.01*,0		0,0.01*,0.01*	1,0.28,0.4		
28 (SS)			0.01*,0.01*		0.99,0.99	

EES day shown with stool 1, stool 2 and cell culture isolate (SSI), if present. Variant and associated amino acid change indicated, if applicable. Blank cells = variant not detected in stool or isolate. *Q<30 for variant in this replicate**.** NGS pipeline reports the variants as SNPs. Coding impact assumes changes are not in common genomes when multiple variants are observed in VP1-143 within the same sample. Paralysis rates are indicated for the samples that have mTgmNVT results available. Grey cells = mTgmNVT result not available.

Supplemental Table 10. *M4a nOPV2-c2.* Frequency of variants associated with amino acid changes in other regions of candidate

EES_day (Samples)	CpG2-3_Cassette				3A	% Paralysis (mice paralyzed/total mice)
	**VP4 G868A A41T**	**VP4 C869T A41V**	**VP3 A1997G H77R**	**VP1 T3016A S179T**	**A5120G Q4R**	
2 (SSI)	0.05,0.02,0.97	0.3,0.37,0.03	0.08,0.12,0.02			0.0 (0/30)
3 (SSI)		0.09,0.08,0				14.3 (4/28)
4 (SS)	0.39,0	0,0.6				
8 (SSI)		0,0.2,0.5	0,0,0.51			
9 (SSI)		0.99,0.28,0.41				
28 (SS)		0.99,0.99	0.96,0.96	0.99,0	0.99,0	

EES day shown with stool 1, stool 2 and cell culture isolate (SSI), if present. Variant and associated amino acid change indicated, if applicable. Blank cells = variant not detected in stool or isolate. *Q<30 for variant in this replicate**.** Paralysis rates are indicated for the samples that have mTgmNVT results available. Grey cells = mTgmNVT result not available.

Supplemental Table 11. *M4 nOPV2-c2.* Frequency of variants in EES at known attenuation sites

EES_day (Samples)	Participant vaccination background	CpG2-3_Cassette, VP1-143		% Paralysis (mice paralyzed/total mice)
		T2909A I143N	T2909C I143T	
4 (SSI)	IPV			0.0 (0/18)
5 (SSI)	OPV			5.3 (1/19)
5 (SSI)	OPV			0.0 (0/20)
6 (SSI)	OPV			0.0 (0/20)
7 (SSI)	OPV			0.0 (0/20)
7 (SSI)	OPV			5.0 (1/20)
8 (SI)	IPV	0.08,0.01	0,0.15	10.0 (1/10)
9 (SS)	IPV			
9 (SSI)	IPV	0.16,0.25,0.02	0,0.01,0	0.0 (0/20)
10 (SSI)	IPV			

EES day shown with stool 1, stool 2 and cell culture isolate (SSI), if present. Variant and associated amino acid change indicated, if applicable. Blank cells = variant not detected in stool or isolate. *Q<30 for variant in this replicate**.** NGS pipeline reports the variants as SNPs. Coding impact assumes changes are not in common genomes when multiple variants are observed in VP1-143 within the same sample. Paralysis rates are indicated for the samples that have mTgmNVT results available. Grey cells = mTgmNVT result not available.

Supplemental Table 12. *M4 nOPV2-c2.* Frequency of variants associated with amino acid changes in other regions of candidate

EES_day (Samples)	Participant vaccination background	CpG2-3_Cassette			2A		3C-pol	% Paralysis (mice paralyzed/total mice)
		VP4 C869T A41V	VP3 G2401A G212S	VP3 CA2418C N218M	C3523T L47F	T3592C Y70H	G5722GA T97N	
4 (SSI)	IPV	0.11,0.22,0	0.24,0.22,0.08		0.01,0,0	0,0,0.02		0.0 (0/18)
5 (SSI)	OPV		0.59,0.6,0.34					5.3 (1/19)
5 (SSI)	OPV							0.0 (0/20)
6 (SSI)	OPV		0.99,0.99,0.99					0.0 (0/20)
7 (SSI)	OPV	0.22,0,0.38	0.03,0,0	0,0.99,0	0.4,1,0.16		0,0.99,0	0.0 (0/20)
7 (SSI)	OPV					0.78,0.77,0.82		5.0 (1/20)
8 (SI)	IPV	0.12,0.14						10.0 (1/10)
9 (SS)	IPV	0.39,0.67						
9 (SSI)	IPV	0.07,0.1,0.25	0.2,0.12,0.05					0.0 (0/20)
10 (SSI)	IPV	0,0.16,0.11	0.26,0.12,0.06					

EES day shown with stool 1, stool 2 and cell culture isolate (SSI), if present. Variant and associated amino acid change indicated, if applicable. Blank cells = variant not detected in stool or isolate. *Q<30 for variant in this replicate**.** Paralysis rates are indicated for the samples that have mTgmNVT results available. Grey cells = mTgmNVT result not available.

Information on Tg66 mice used in Figure 3 and Table 1:

The male founder animal was generated by pronuclear injection of the human genomic DNA insert of cosmid PRG-1 (*Ren RB, Costantini F, Gorgacz EJ, Lee JJ, Racaniello VR. Transgenic mice expressing a human poliovirus receptor: a new model for poliomyelitis. Cell. 1990;63(2):353-362*) into (C57BL/6 x CBA/J) F2 zygotes. The PVR gene is stably integrated into a single locus in the mouse genome without segregation distortion. Tg66-CBA mice are the product of crossing Tg66 with CBA/J mice. The mice are homozygous for PVR and class II I-Aβ genes (H2k).

The Tg66-CBA mice are readily available at NIBSC, where much of the preclinical work to characterize the nOPV2 strains was conducted. This mouse line is used in *Knowlson S, Burlison J, Giles E, Fox H, Macadam AJ, Minor PD. New Strains Intended for the Production of Inactivated Polio Vaccine at Low-Containment After Eradication. PLoS Pathog. 2015 Dec 31;11(12).* The mice were used in substitution for the Tg21 strain used in the WHO neurovirulence assay to evaluate vaccine strains. The data show that the Tg66 mouse model distinguishes between wild type and vaccine strains, and that the mice are highly sensitive to mutations in domain V that are involved in the attenuation phenotype. Both mouse strains (Tg66 and Tg21) have similar sensitivities to Sabin viruses when inoculated by the intraspinal route (unpublished data).

The Tg66-CBA mouse line was used to evaluate the neurovirulence of the novel OPV type 2 strains. The results of the evaluation of the two nOPV2 candidates using these mice are published as indicated below:

Konopka-Anstadt JL, Campagnoli R, Vincent A, et al. Development of a new oral poliovirus vaccine for the eradication end game using codon deoptimization. NPJ Vaccines. 2020;5:26. Published 2020 Mar 20. doi:10.1038/s41541-020-0176-7

Yeh MT, Bujaki E, Dolan PT, et al. Engineering the Live-Attenuated Polio Vaccine to Prevent Reversion to Virulence. Cell Host Microbe. 2020;27(5):736-751.e8. doi:10.1016/j.chom.2020.04
