# Supplementary material for: Assessment of genetic changes and neurovirulence of shed Sabin and novel type 2 oral polio vaccine viruses
Source: NPJ Vaccines. 2021 Jul 29;6:94. doi: 10.1038/s41541-021-00355-y (PMC8322168; doi:10.1038/s41541-021-00355-y)
Supplement: Supplementary file 2 — Reporting Summary [file 41541_2021_355_MOESM2_ESM.pdf]

nature portfolio

Corresponding author(s): Rahnuma Wahid

Last updated by author(s): Jun 9, 2021

Reporting Summary

Nature Portfolio wishes to improve the reproducibility of the work that we publish. This form provides structure for consistency and transparency in reporting. For further information on Nature Portfolio policies, see our Editorial Policies and the Editorial Policy Checklist.

Statistics

For all statistical analyses, confirm that the following items are present in the figure legend, table legend, main text, or Methods section.

n/a	Confirmed	
☐	☒	The exact sample size (*n*) for each experimental group/condition, given as a discrete number and unit of measurement
☐	☒	A statement on whether measurements were taken from distinct samples or whether the same sample was measured repeatedly
☒	☐	The statistical test(s) used AND whether they are one- or two-sided<br>*Only common tests should be described solely by name; describe more complex techniques in the Methods section.*
☒	☐	A description of all covariates tested
☒	☐	A description of any assumptions or corrections, such as tests of normality and adjustment for multiple comparisons
☐	☒	A full description of the statistical parameters including central tendency (e.g. means) or other basic estimates (e.g. regression coefficient) AND variation (e.g. standard deviation) or associated estimates of uncertainty (e.g. confidence intervals)
☒	☐	For null hypothesis testing, the test statistic (e.g. *F*, *t*, *r*) with confidence intervals, effect sizes, degrees of freedom and *P* value noted<br>*Give P values as exact values whenever suitable.*
☒	☐	For Bayesian analysis, information on the choice of priors and Markov chain Monte Carlo settings
☒	☐	For hierarchical and complex designs, identification of the appropriate level for tests and full reporting of outcomes
☐	☒	Estimates of effect sizes (e.g. Cohen's *d*, Pearson's *r*), indicating how they were calculated

Our web collection on statistics for biologists contains articles on many of the points above.

Software and code

Policy information about availability of computer code

Data collection	For next-generation sequencing: MiSeq instrument with analysis software version 1.8.46 (Illumina)
Data analysis	R version 4.0.31; Proprietary algorithm at Viroclinics comprised of trimming (Trimgalore version 0.4.4), mapping (BWA mem; version 0.7.16a-r1181), and SNP (single nucleotide polymorphism) variant calling where multinucleotide polymorphisms and COMPLEXES are split into multiple SNPs using the vcfallelicprimitives utility from vcflib library (freebayes; version v1.1.0-60-gc15b070)

For manuscripts utilizing custom algorithms or software that are central to the research but not yet described in published literature, software must be made available to editors and reviewers. We strongly encourage code deposition in a community repository (e.g. GitHub). See the Nature Portfolio guidelines for submitting code & software for further information.

Data

Policy information about availability of data

All manuscripts must include a data availability statement. This statement should provide the following information, where applicable:

- Accession codes, unique identifiers, or web links for publicly available datasets
- A description of any restrictions on data availability
- For clinical datasets or third party data, please ensure that the statement adheres to our policy

Datasets specific to this publication are available from the corresponding author upon reasonable request and contingent on the requested uses being permitted under the informed consent received from the source clinical study, where applicable.
The full genome sequence of nOPV2 candidate 1 can be found in GenBank (accession number MZ245455). The full genome sequence of nOPV2 candidate 2 can be found in GenBank (accession number MN654096). Sequencing data can be accessed on the SRA database (accession number PRJNA735689).

Field-specific reporting

Please select the one below that is the best fit for your research. If you are not sure, read the appropriate sections before making your selection.

☒ Life sciences ☐ Behavioural & social sciences ☐ Ecological, evolutionary & environmental sciences

For a reference copy of the document with all sections, see nature.com/documents/nr-reporting-summary-flat.pdf

Life sciences study design

All studies must disclose on these points even when the disclosure is negative.

Sample size	Trials were not powered with consideration of the next-generation sequencing (NGS) and neurovirulence (NV) analyses, as these results support exploratory aims of the trial study protocol. Every sample meeting the criteria for exploratory endpoint specimen (EES) was tested by NGS/NV test.
Data exclusions	Selection of EES was conducted as described in the manuscript. No EES were excluded from testing unless insufficient material was available. All available data meeting pre-specified criteria are reported for both next-generation sequencing and the mouse neurovirulence test.
Replication	Majority of the EES were tested in separate mouse neurovirulence tests at least twice and results are reproducible. All EES stools were assessed in duplicate and variant profiles were generally reproducible.
Randomization	In the mouse neurovirulence (NV) test, randomization is required for the mouse to sample/control dose, cage locations and order of inoculations. Randomization was conducted per current SOPs at the testing facility. Samples for the NV test and next-generation sequencing were not formally randomized to test, but all samples were blinded (see below).
Blinding	All test samples (EES) were de-identified (no participant data was associated with the test samples). Investigators were blinded to all sample associated information (group allocation data). Additional blinding of samples was applied prior to conduct of the mouse neurovirulence test or next-generation sequencing.

Reporting for specific materials, systems and methods

We require information from authors about some types of materials, experimental systems and methods used in many studies. Here, indicate whether each material, system or method listed is relevant to your study. If you are not sure if a list item applies to your research, read the appropriate section before selecting a response.

Materials & experimental systems

n/a	Involved in the study	
☒	☐	Antibodies
☐	☒	Eukaryotic cell lines
☒	☐	Palaeontology and archaeology
☐	☒	Animals and other organisms
☒	☐	Human research participants
☒	☐	Clinical data
☒	☐	Dual use research of concern

Methods

n/a	Involved in the study	
☒	☐	ChIP-seq
☒	☐	Flow cytometry
☒	☐	MRI-based neuroimaging

Eukaryotic cell lines

Policy information about cell lines

Cell line source(s)	HEp-2C (ATCC, CCL-23)
Authentication	Cell line was not authenticated
Mycoplasma contamination	Cell line tested negative for mycoplasma
Commonly misidentified lines (See ICLAC register)	Not applicable

Animals and other organisms

Policy information about studies involving animals; ARRIVE guidelines recommended for reporting animal research

Laboratory animals	Poliovirus receptor transgenic mice - Tg66 and Tg21 lines were used. Both male and female mice between the ages of 6 to 8 weeks.

Wild animals	Study did not involve wild animals
Field-collected samples	Study did not involve samples collected from the field
Ethics oversight	The Tg21 mouse experiments at Viroclinics were conducted in compliance with Dutch Animal Testing Act (WOD) and according to the DIRECTIVE 2010/63/EU of The European Parliament and of the Council of 22 September 2010 on the protection of animals used for scientific purposes under project license 2770020171404 issued by the central authorizing body (Centrale Commissie Dierproeven). Ethical approval for the study was obtained in working protocols 1-3 from the animal ethics committee (Instantie voor Dierenwelzijn) under the above project license.<br>The Tg66 mouse experiments at NIBSC were performed under licenses PPL 80/2478 and PPL 70/8979 granted by the UK Home Office under the Animal (Scientific Procedures) Act 1986 revised 2013 and reviewed by the internal NIBSC Animal Welfare and Ethics Review Board.

Note that full information on the approval of the study protocol must also be provided in the manuscript.
